# Prehospital qSOFA, mSOFA, and NEWS2 performance for sepsis prediction: A prospective, multi-center, cohort study

**DOI:** 10.3389/fmed.2023.1149736

**Published:** 2023-04-18

**Authors:** Laura Melero-Guijarro, Ancor Sanz-García, Francisco Martín-Rodríguez, Vivian Lipari, Cristina Mazas Perez Oleaga, Stefanía Carvajal Altamiranda, Nohora Milena Martínez López, Irma Domínguez Azpíroz, Miguel A. Castro Villamor, Irene Sánchez Soberón, Raúl López-Izquierdo

**Affiliations:** ^1^Emergency Department, Complejo Asistencial Universitario de Palencia, Palencia, Spain; ^2^Faculty of Health Sciences, Universidad de Castilla la Mancha, Talavera de la Reina, Spain; ^3^Prehospital Early Warning Scoring-System Investigation Group, Valladolid, Spain; ^4^Faculty of Medicine, Universidad de Valladolid, Valladolid, Spain; ^5^Advanced Life Support, Emergency Medical Services (SACYL), Valladolid, Spain; ^6^Universidad Europea del Atlántico, Santader, Spain; ^7^Universidad Internacional Iberoamericana, Campeche, Mexico; ^8^Universidad Internacional Iberoamericana, Arecibo, PR, United States; ^9^Universidade Internacional do Cuanza, Cuito, Bié, Angola; ^10^Fundación Universitaria Internacional de Colombia, Bogotá, Colombia; ^11^Universidad de La Romana, La Romana, Dominican Republic; ^12^Emergency Department, Hospital Universitario Rio Hortega, Valladolid, Spain

**Keywords:** sepsis, early warning scores, septic shock, point-of-care testing, mSOFA, prehospital

## Abstract

**Background:**

Nowadays, there is no gold standard score for prehospital sepsis and sepsis-related mortality identification. The aim of the present study was to analyze the performance of qSOFA, NEWS2 and mSOFA as sepsis predictors in patients with infection-suspected in prehospital care. The second objective is to study the predictive ability of the aforementioned scores in septic-shock and in-hospital mortality.

**Methods:**

Prospective, ambulance-based, and multicenter cohort study, developed by the emergency medical services, among patients (*n* = 535) with suspected infection transferred by ambulance with high-priority to the emergency department (ED). The study enrolled 40 ambulances and 4 ED in Spain between 1 January 2020, and 30 September 2021. All the variables used in the scores, in addition to socio-demographic data, standard vital signs, prehospital analytical parameters (glucose, lactate, and creatinine) were collected. For the evaluation of the scores, the discriminative power, calibration curve and decision curve analysis (DCA) were used.

**Results:**

The mSOFA outperformed the other two scores for mortality, presenting the following AUCs: 0.877 (95%CI 0.841–0.913), 0.761 (95%CI 0.706–0.816), 0.731 (95%CI 0.674–0.788), for mSOFA, NEWS, and qSOFA, respectively. No differences were found for sepsis nor septic shock, but mSOFA’s AUCs was higher than the one of the other two scores. The calibration curve and DCA presented similar results.

**Conclusion:**

The use of mSOFA could provide and extra insight regarding the short-term mortality and sepsis diagnostic, backing its recommendation in the prehospital scenario.

## Highlights

- mSOFA allows the prehospital early identification of high-risk sepsis patients.
- mSOFA presents a good predictive power for short-term mortality of Sepsis patients.
- mSOFA allows sepsis and septic shock diagnosis.

## Introduction

Timely characterization of severity in patients with suspected infection is a critical step in order to improve survival. A well-known long-standing point is that sepsis and septic shock are life-threatening diseases, early detection of the associated organ dysfunction is critical to start the therapeutic measures as soon as possible (1). In this regard, the use of early warning scores (EWS) is playing an important role in the quick detection of high-risk patients (2, 3).

Currently, sepsis is typically defined as a multi-organ dysfunction of infectious origin presenting a significant morbidity-mortality, which it is exponentially enhanced with septic shock. Since 2016, EWS utilization has been promoted and generalized both for suspicion and diagnosis of organ failure. Being the Sequential Organ Failure Assessment (SOFA) score the most known (4–6). SOFA score assesses multi-organ failure by aggregate points based on analytical, hemodynamic, and physiological parameters. However, in prehospital care, due to the impossibility to obtain all the required variables, the recommendation for detecting sepsis in an infected or suspected infected patient is based on the use of the quick sequential organ failure assessment (qSOFA) score (7). Following the publication of the Surviving Sepsis Campaign Guidelines in October 2021, the use of the qSOFA score for the early detection of sepsis has been questioned, and the guidelines suggest alternative EWS, such as, the National Early Warning Score 2 (NEWS2) (8). According to recent studies, NEWS2 reported better sensitivity and discrimination of critically ill patients than qSOFA score (9, 10).

In prehospital care, therefore, in the recent past, initial screening of sepsis required suspicion of infection and application of EWS, but thanks to the incorporation of point-of-care laboratory testing (POCT), basic analytical results are available bedside (11, 12). POCT offers an increasing range of biomarkers, reporting results few minutes after the test, and is becoming an increasingly widespread diagnostic resource in Emergency Medical Services (EMS) (13). Based on POCT, new scoring systems have been derived and validated, adding analytical parameters (e.g., lactate, creatinine, urea, ions, venous blood gases) to the classically employed physiological endpoints, improving prediction of clinical outcomes (14–16). Similarly to the SOFA score, the modified Sequential Organ Failure Assessment (mSOFA) was developed using parameters available in prehospital care: the pulse oximetry saturation/fraction of inspired oxygen ratio (SaFi), mean arterial pressure (MAP), creatinine, Glasgow Coma Scale scale (GCS) and lactate, making mSOFA an ideal scoring system for EMS purposes (17).

We aimed to analyze the performance of qSOFA, NEWS2 and mSOFA as sepsis alarm-triggers in patients with infection-suspected in prehospital care. Secondly, the feasibility by these scoring systems to predict sepsis-related worst outcomes: septic-shock and in-hospital mortality.

## Methods

### Study design

This is a prospective, ambulance-based, and multicenter cohort study, developed by the EMS, among patients with suspected infection transferred by ambulance with high-priority to the emergency department (ED).

The study enrolled six advanced life support units (ALSU), 38 basic life support units (BLSU) and four ED in the provinces of Salamanca, Segovia, and Valladolid (Spain), with a total referral population of 995,137 residents, between 1 January 2020 and 30 September 2021. All the facilities were managed by the Castilla y León Public Health System (SACYL). Basic life support units (BLSU) are made up of two emergency medical technicians (EMT), and advanced life support units (ALSU) include two EMT, an emergency registered nurse (ERN) and a physician, performing standard life support procedures according to protocols on-scene or *en route*.

### Population

Adult patients (age ≥ 18 years), with suspected infection and presenting acute illness, evaluated by ALSU and transferred by ambulance (ALSU or BLSU) to the ED were eligible. Suspected infection was considered by the ALSU team based on clinical history and anamnesis, evidence of febrile syndrome, and/or related signs and symptoms. On-scene non-recovery cardio-respiratory arrest, end-stage patients, pregnant women, acute psychiatric disorders, situations with on-scene risk for the staff, on-site discharge (upon ALSU-physician evaluation), and cases without informed consent were excluded. Likewise, cases were also excluded when intravenous access was impossible, making prehospital analysis and the subsequent calculation of the mSOFA unfeasible. Therefore, we present here a complete-case study, where no missing data were allowed.

### Outcomes

The primary outcome was in-hospital sepsis diagnosis. To ensure uncontroversial results, the Third International Consensus Definitions for Sepsis and Septic Shock (Sepsis-3) (4, 5) and the guidelines of the Surviving Sepsis Campaign Guidelines (8) were followed, in particular, the clinical situation of sepsis, was considered for all patients with suspected infection and a SOFA score of 2 or more points. As a secondary outcome, the diagnostic of septic-shock, and 2-day in-hospital mortality (all causes but with sepsis diagnosis) were recorded, such a specific time window for mortality was selected to evaluate the short-term prognostic accuracy of the score, in such a way that the cause of death was linked to the condition that motivated the transfer, rather than linked to in-hospital events. Septic-shock was defined as a patient with sepsis that, following volume resuscitation (20 mL/kg), required vasoactive drug support to maintain MAP over 65 mmHg and presented a lactate level over 2 mmol/L.

### Data collection

Socio-demographic data (age, sex, and nursing home residence), standard vital signs (respiratory rate, oxygen saturation, systolic and diastolic blood pressure, heart rate, temperature and GCS) were collected by the ERN during first patient contact, i.e., from prehospital setting. Pulse oxygen saturation, blood pressure and pulse, were determined with the LifePAK® 15 monitor-defibrillator (Physio-Control, Inc., Redmond, United States), and temperature with the ThermoScan® PRO 6000 thermometer (Welch Allyn, Inc., Skaneateles Falls, United States). Following this, a prehospital analysis was performed with the epoc® Blood Analysis System (Siemens Healthcare GmbH, Erlangen Germany), providing, in particular, glucose, lactate and creatinine values (18, 19).

Subsequently, SaFi (pulse saturation/inspired oxygen fraction ratio) and MAP [systolic blood pressure + 2 (diastolic blood pressure)/3] were calculated, in order to calculate the analyzed scores. The qSOFA considers respiratory rate, systolic blood pressure and level of consciousness (assumed as abnormal GCS different from 15 points) (6). NEWS 2 is composed by respiratory rate, oxygen saturation, oxygen support, systolic blood pressure, heart rate, temperature, and level of consciousness (assumed as abnormal GCS different from 15 points) (20). Finally, the mSOFA required SaFi, MAP, GCS, creatine, and lactate for calculation (17).

After a 2-day follow-up period, an associate researcher, by reviewing the electronic medical record, collected the hospital outcomes: diagnosis of sepsis and septic-shock, comorbidities, hospital-inpatient, intensive care unit-admission, mechanical ventilation, norepinephrine use and 2-day in-hospital mortality.

### Data analysis

The quantitative variables presented non-normal distributions (assessed by Shapiro–Wilk and Lilliefors tests); therefore, quantitative variables were described as median and interquartile range (25th–75th percentile), and for comparison purposes, the Mann–Whitney *U*-test was used. Absolute frequencies and 95% confidence interval (95%CI) were used to describe categorical variables, and the Chi-square test were used for 2 × 2 contingency tables or/and contrast of proportions, if necessary, by using (percentage of cells with expected values less than five, greater than 20%) Fisher’s exact test was used.

All statistical analyses were performed using our own codes and base functions in R, version 4.1.2.^1^

### Validity and reliability

The following process was replicated for each outcome considered in this study. Firstly, the three scores were calculated and then three different methods were used to compare their performance:

1. The discrimination capacity of each score was performed by fitting the model in a generalized linear model. The model included the outcome and the score (this process was repeated for each score). Then, we determined the AUC of the receiver operating characteristic (ROC), a *p*-value of the hypothesis test (H0: ABC = 0.5), and its corresponding CI95%. Likewise, further statistical characteristics as: the global sensitivity, specificity, positive predictive value, negative predictive value, positive likelihood ratio, negative likelihood ratio and the optimal sensitivity and specificity (from the Youden point) were also determined. The resulting AUCs, from each score, were compared by using the Delong’s test.
2. The calibration curve of each score was assessed by plotting observed vs. predicted patients. From that curve further indexes and statistics were computed to allow their comparison.
3. The decision curve of each score was computed by representing the net benefit against the threshold probability.

### Ethical considerations

Patients were collected from two back-to-back prospective studies carried out under the identical operative guideline. The institutional review board of Public Health Service reviewed and approved the investigation (reference: PI-049-19/PI-GR-19-1258). The study protocol was registered in the WHO International Clinical Trials Registry Platform (ISRCTN48326533 and ISRCTN49321933); we followed the Transparent reporting of a multivariable prediction model for individual prognosis or diagnosis (TRIPOD) (21) guidelines (Supplementary Data P1). Written informed consent was obtained from all the study participants. Patients without informed consent were excluded.

## Results

**Figure 1:**
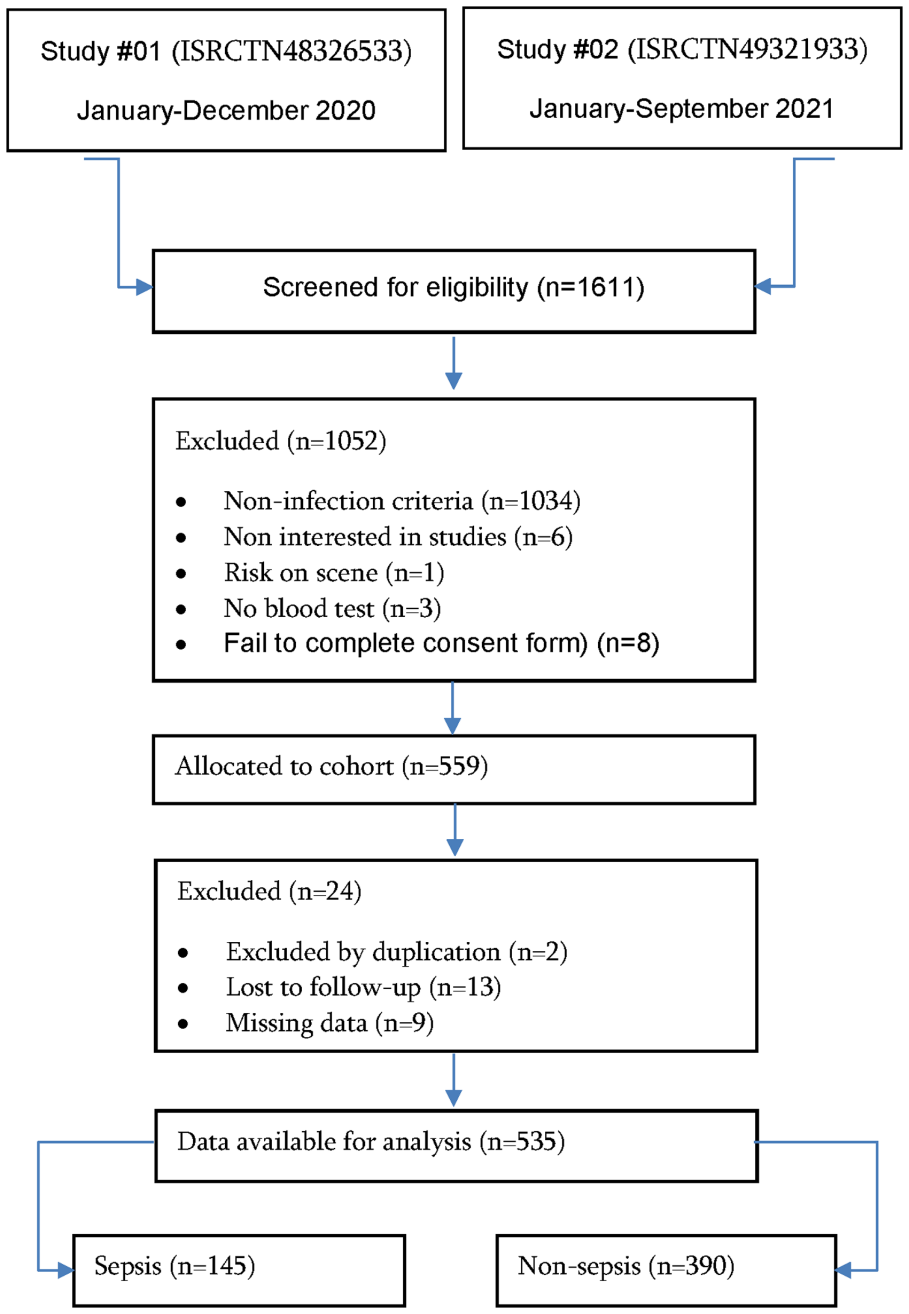
Flowchart study population.

Overall, 535 patients with suspected infection were included in the follow-up study (see Figure 1). The median age was 76 years (IQR: 64–85 years), 39.3% were female (210 cases), and the rate of institutionalized patients was 26.9% (144 cases). The in-hospital rate was 81.5% (436 cases), resulting in a clinical diagnosis of sepsis of 27.1% (145 cases; Table 1), a septic-shock rate of 6.7% (36 cases), and 2-day in-hospital mortality of 14.2% (76 cases) (in Supplementary Tables S1, S2 patient characteristics according of septic-shock and 2-day in-hospital mortality can be observed).

**Table 1:** Baseline patients’ characteristics according sepsis vs. non-sepsis cases.

	Total	Sepsis	Non-sepsis	*p-*value^b^
No. (%) with data^a^	535	145 (27.1)	390 (72.9)	
Age, year	76 (64–85)	77 (66–86.5)	75 (63–85)	0.135
Sex, Female	210 (39.3)	53 (25.2)	157 (74.8)	0.435
Nursing homes	144 (26.9)	54 (37.5)	90 (62.5)	0.001
**Prehospital vital signs**				
RR (breaths/min)	26 (19–32)	28 (24.5–33)	25 (18–32)	0.002
SpO2 (%)^c^	92 (86–96) [91.5 ± 2.8]	92 (82–94.5) [90 ± 3.4]	93 (87–96) [92.2 ± 2.5]	0.005
FiO2 (%)^c^	21 (21–24) [21 ± 0.8]	21 (21–26) [22.2 ± 1.4]	21 (21–21) [21 ± 0.2]	0.002
SaFi	428.6 (342.8–457.1)	391.6	430.9 (357.6–457.1)	<0.0001
SBP (mmHg)	131 (104–150)	104 (86.5–134.5)	135 (113.75–153)	<0.0001
DBP (mmHg)	72 (60–87)	61 (50–79)	76 (64–89)	<0.0001
MBP (mmHg)	93 (76.3–107.3)	75.34 (61.6–96.5)	95.6 (81.9–110)	<0.0001
Heart rate (beats/min)	98 (80–117)	105 (83.5–124)	97 (79–114.25)	0.006
Temperature (°C)	36.7 (36.1–37.9)	37.6 (36.2–38.65)	36.7 (36.1–37.6)	<0.0001
GCS (points)	15 (14–15)	14 (11–15)	15 (15–15)	<0.0001
**Prehospital biomarkers**				
Glucose, mg/dL	144 (114–201)	162 (120–220)	141 (113–192.25)	0.018
Lactate, mmol/L	2.64 (1.76–3.80)	3.69 (2.85–5.80)	2.13 (1.47–3.10)	<0.0001
Creatinine, mgr/dL	1.14 (0.83–1.78)	1.76 (1.13–2.6)	1.05 (0.77–1.45)	<0.0001
**Prehospital scores**				
qSOFA, points	1 (1–2)	2 (1–3)	1 (0–1)	<0.0001
NEWS2, points	8 (5–10)	11 (8–13)	7 (4–9)	<0.0001
mSOFA, points	3 (1–6)	6 (3–9)	2 (1–4)	<0.0001
Hospital outcomes				
CACI, points	6 (5–9)	7 (5–9)	6 (4–9)	0.042
Hospital-inpatient	436 (81.5)	144 (33.0)	292 (67.0)	<0.0001
ICU-admission	69 (12.9)	39 (56.5)	30 (43.5)	<0.0001
Mechanical ventilation	62 (11.6)	26 (41.9)	36 (58.1)	0.005
Norepinephrine	79 (14.8)	48 (69.8)	31 (39.2)	<0.0001
Septic shock	36 (6.7)	36 (100)	0 (0)	<0.0001
In-hospital mortality	76 (14.2)	76 (100)	0 (0)	<0.0001

RR, respiratory rate; SPO2, oxygen saturation; FIO2, fraction of inspired oxygen; SaFi, pulse oximetry saturation/fraction of inspired oxygen ratio; SBP, systolic blood pressure; DBP, diastolic blood pressure; MBP, mean blood pressure; HR, heart rate; GCS, Glasgow coma scale; qSOFA, quick sequential organ failure assessment; NEWS2, National Early Warning Score 2; mSOFA, modified Sequential Organ Failure Assessment; ACCI, Adjusted age-adjusted Charlson comorbidity index; ICU, intensive care unit.

a Values expressed as total number (fraction) and medians (25–75 percentiles), as appropriate.

b The Mann–Whitney *U*-test or Chi-squared test was used as appropriate.

c The mean and standard deviation is shown between square brackets due to the similarity between medians.

**Figure 2:**
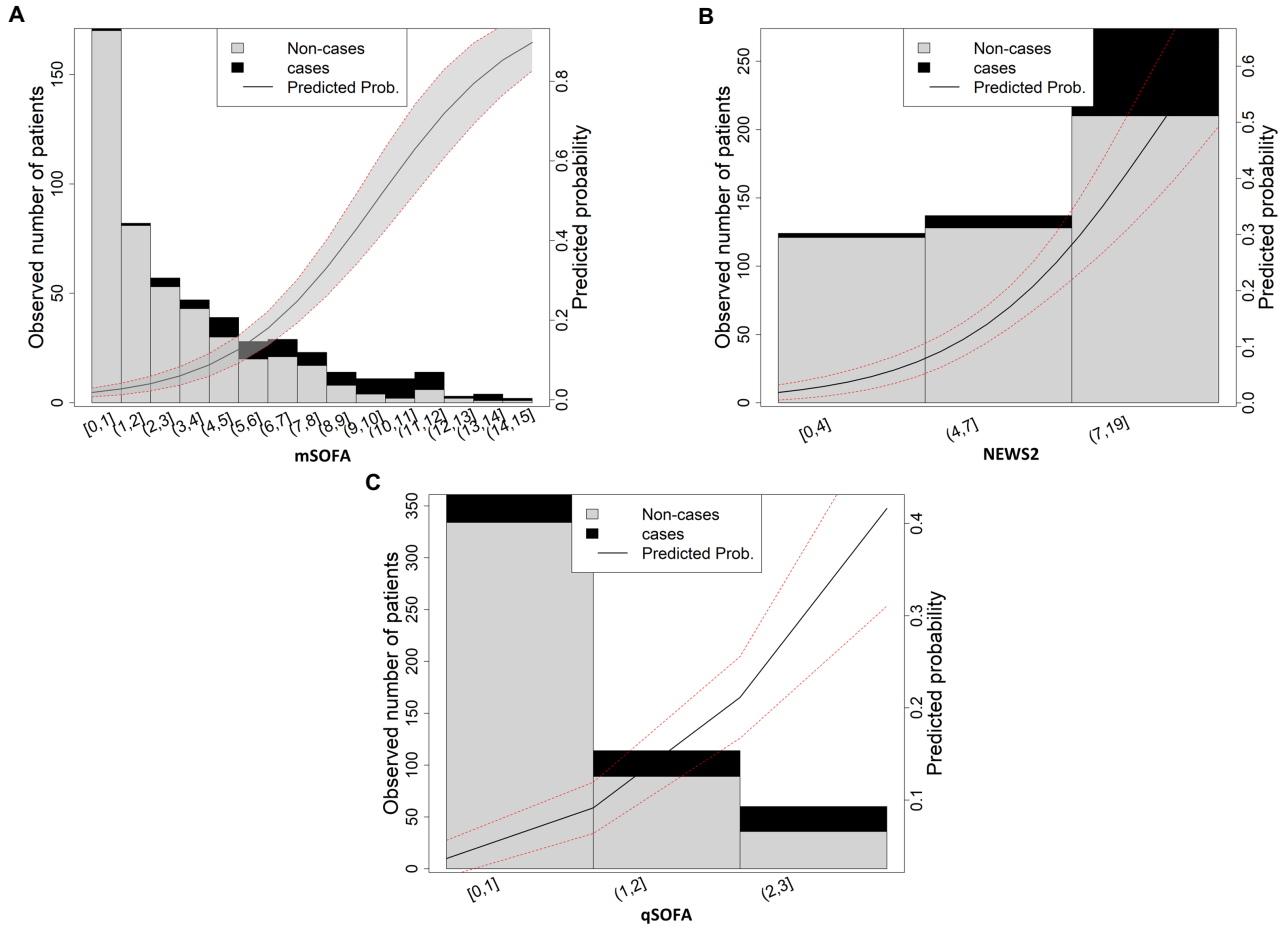
Scores values vs. real and predicted probability for mortality **(A)** mSOFA, **(B)** NEWS2, **(C)** qSOFA. The solid line shows the predicted probability of the outcome; area between dashed lines shows the 95% confidence interval.

The representation of score values against the observed number of patients for mortality (Figure 2) showed that higher levels of score values mean higher mortality rates. When comparing the maximum predicted probability of mortality of each score, mSOFA reached a > 80%, NEWS2 > 60%, and qSOFA a > 40% in the highest values of each score. This was not the case for sepsis (Supplementary Figure S1), since all scores presented similar percentages of predicted probability (80%). Finally for septic shock, the predicted probability was similar for mSOFA and NEWS2 (50 and 40% respectively), but not for qSOFA, which presented a 25%.

**Figure 3:**
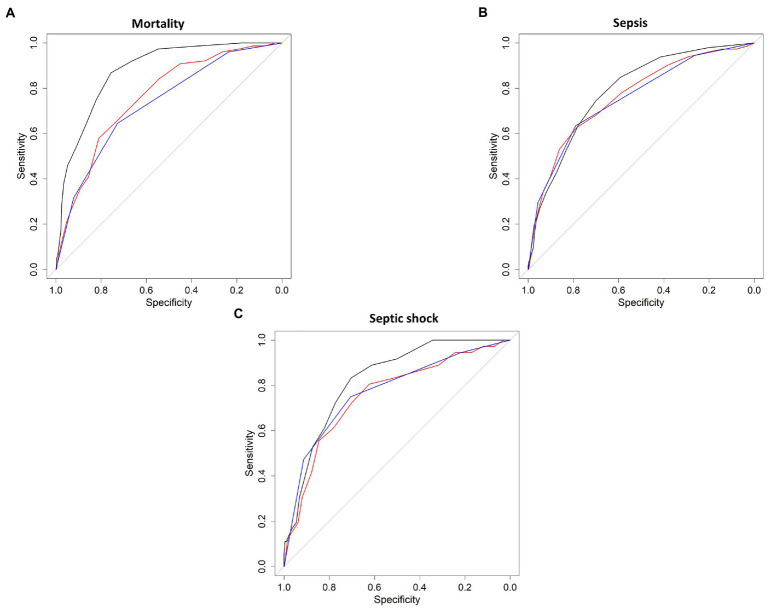
Discrimination capacity of each model. Solid line shows the receiver operating characteristic (ROC) curve for mortality **(A)**, sepsis **(B)**, and septic shock **(C)**. black line = mSOFA, red line = NEWS2, blue lines = qSOFA.

The discrimination capacity of each score for all the outcomes is shown in Figure 3, one on one comparison of each result with *p* values is shown in Supplementary Table S3. Only the comparison between scores for mortality outcome presented statistically significant differences, as can be observed in Figure 3A. The mSOFA outperformed the other two scores (*p* < 0.001 vs. both scores), presenting the following AUCs: 0.877 (95%CI 0.841–0.913); *p* < 0.001, 0.761 (95%CI 0.706–0.816); *p* < 0.001, 0.731 (95%CI 0.674–0.788); *p* < 0.001, for mSOFA, NEWS, and qSOFA, respectively. Despite mSOFA presented higher AUCs, no statistically significant differences were found for sepsis (Figure 3B) nor septic shock (Figure 3C), presenting AUCs that ranged between 0.825 and 0.755 (Supplementary Table S3). These results were also similar for the parameters derived from ROC curves (Supplementary Table S4).

**Figure 4:**
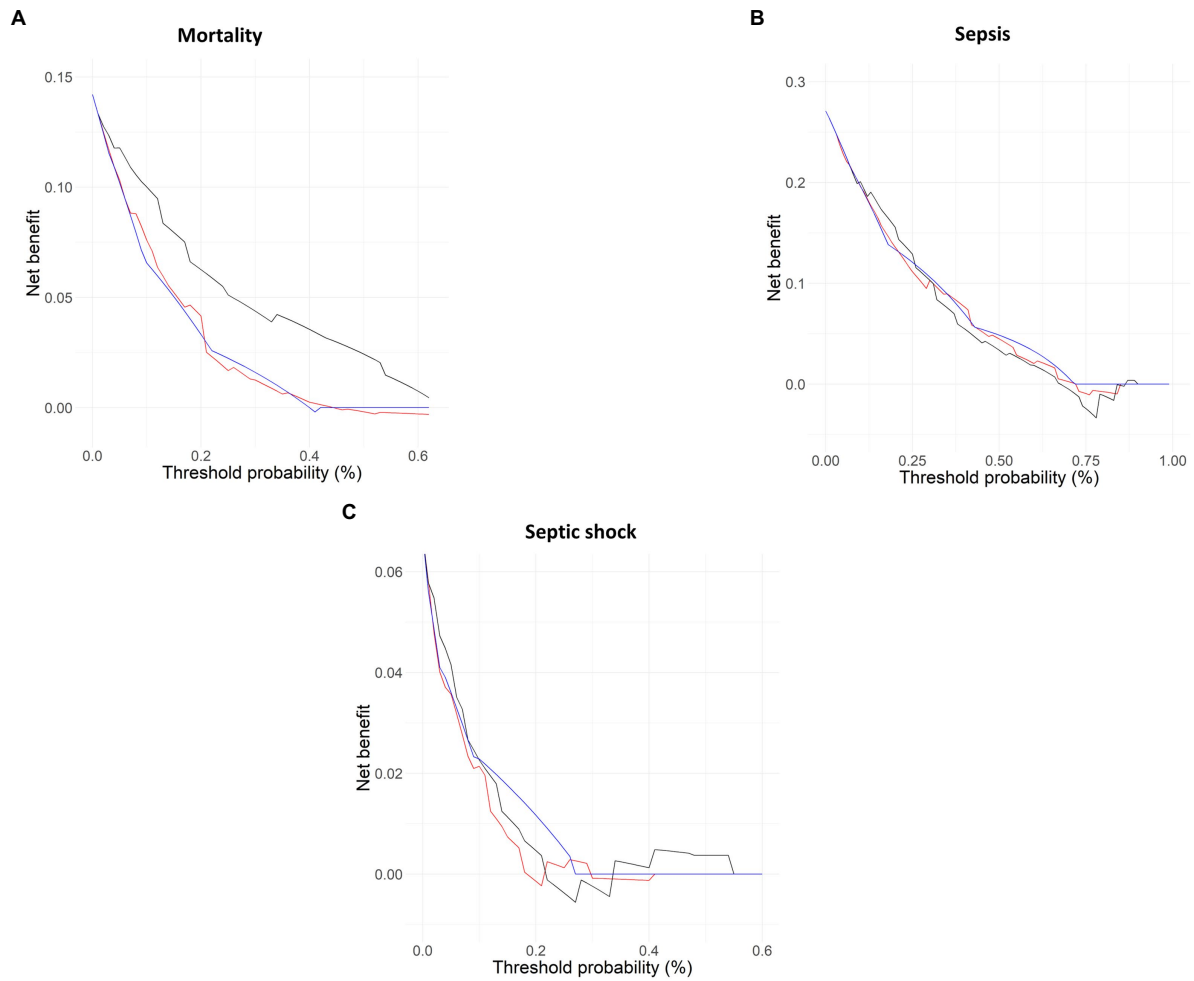
Decision curve analysis for each model. Solid line shows the decision (ROC) curve for mortality **(A)**, sepsis **(B)**, and septic shock **(C)**. black line = mSOFA, red line = NEWS2, blue lines = qSOFA.

The decision curve analysis (Figure 4) confirmed the aforementioned AUC results. Mortality was the only outcome for which the scores presented differences. Figure 4A shows that mSOFA presented a higher net benefit as compared to the other two scores for all the threshold probability. This was not the case for the other two outcomes, sepsis, and septic shock, for which no differences were found (Figures 4B,C).

Lastly, calibration curve, i.e., the result derived from representing the observed vs. the predicted probability also confirmed the above results. The calibration curve for mortality (Supplementary Figure S2A) showed that, despite both intercept and slope were identical, brier score (a representation of how accurate the predictions are, low values mean highest accuracy) was lower for mSOFA. No differences were found for the other two outcomes: sepsis and septic shock (Supplementary Figures S2B,C, respectively).

## Discussion

This study is the first to analyze the usefulness of a novel prehospital mSOFA score to detect sepsis and immediate life-threatening risk in suspected infected patients, and to compare with two of the currently promoted scores for sepsis diagnosis (8). In this regard, our results show that mSOFA performed excellently in identifying 2-day mortality among infected patients, being significantly superior to both qSOFA and NEWS2. However, although the mSOFA score obtains better AUC than the qSOFA and NEWS2 scales for the diagnosis of sepsis and septic shock, these differences were not statistically significant.

At the present time, no studies exist that analyze the usefulness of the prehospital mSOFA described in this study among an infectious population. In the original article, the AUC of the referral cohort for the detection of 2-day mortality was 0.94 (17), somewhat above the result of the present study. This decrease in the predictive ability of the mSOFA score among this group of patients may be attributable to several reasons. Firstly, this score was designed on a population basis that is not specific to infected patients, and secondly, the cardiovascular parameter of the score has a lower weight than the rest of the variables, since the scores derived from the administration of vasoactive drugs were eliminated in the prehospital mSOFA to facilitate use and be more in accordance with the usual clinical practice of EMS. Both reasons may have influenced to reduce prognostic ability among infected patients.

Anyway, we can confirm that, for the analysis of infected patients, the AUC for predicting 2-day mortality and consequently immediate probability risk of death, continues to be good, and exceeds that obtained by both qSOFA and NEWS2 in our study, as well as that obtained by other studies. Dadeh et al. (22) analyzed the predictive capacity of 2-day mortality NEWS and NEWS-Lactate among infected patients and obtained AUCs of 0.79 and 0.81 respectively, again lower than those obtained by mSOFA. On the other hand, if we analyze the predictive capacity of qSOFA and prehospital NEWS for 30-day mortality, the AUCs decrease slightly, as demonstrated by Silock et al. (23) who obtained AUCs of 0.68 for qSOFA and 0.73 for NEWS2, figures much lower than the performance of mSOFA. These data suggest that these early warning scores can be useful in short-term prognostic assessment, which makes them very useful for the management of patients in prehospital care, where early decision making can condition the patient’s life.

As indicated by previous reports, the use of lactate provides a better predictive capacity than the use of scores alone, since this biomarker behaves as an independent mortality factor in septic patients (15, 17, 24–26). Lactate, together with the other parameters that make up the mSOFA, allows the evaluation of tissue hypoperfusion, in addition to being a fundamental biomarker for the current definition of septic shock (5). The use of another biochemical parameter such as creatine allows mSOFA to detect early patients who are developing infection-associated renal failure, in agreement with scores such as CURB-65 (27).

Regarding the diagnosis of sepsis, mSOFA, NEWS and qSOFA behave in a similar way with practically equivalent AUCs. The results obtained are similar to those found in other healthcare settings such as elderly patients where the diagnostic capacity is 0.74 and 0.76 for NEWS and qSOFA, respectively, (28) or ED where the ability to diagnose sepsis among patients at risk of infection is slightly higher for qSOFA (0.79) and similar for NEWS2 (0.85) (29). In a review carried out by Lane et al. (30) to determine the best models for the detection of sepsis in the prehospital setting, they observed that the predictive capacity of qSOFA and NEWS was higher than 0.80, and compared with other models both presented the best performance for diagnosis (30). These data support the current capacity of these EWS for the early recognition of prehospital sepsis.

NEWS2 provides superior results to qSOFA in all studies. This superior performance may be attributable to the composition of the scoring systems: NEWS2 is based on the evaluation of 7 parameters, vs. 3 for qSOFA (31). In contrast, in the present cohort, we found that qSOFA has a superior sensitivity to NEWS2, both for sepsis diagnosis prediction and 2-day in-hospital mortality, contrasting with previous studies in which qSOFA is less sensitive than NEWS2, but more specific (8–10, 23, 29, 30). Our results present a sensitivity for the qSOFA for sepsis of 0.7, as compared to 0.4 reported in Lane et al. (30). The difference observed is probably of new explained by the fact that we analyzed the outcome variable at 2- vs. 30-day in other studies. As a result, one might suggest that qSOFA has a good short-term performance, being, therefore, a useful tool for sepsis screening in the first days after.

The latest Surviving Sepsis Campaign Guidelines place an increased emphasis on the search for alternatives in sepsis detection systems, encouraging the use of different screening methods (8). The present work describes a new score that improve the prognostic and diagnostic capacity of qSOFA and NEWS2. Additionally, the mSOFA performs with a significantly superior sensitivity to the other two scores when the best cut-off point is considered, suggesting a remarkable reliability of mSOFA when used in prehospital care.

Lastly, the analysis by clinical prediction curves confirms that mSOFA score performs consistently better and is able to identify patients with a high-risk of mortality across all probability conditions. This confirms that the modification of current used scores, by either adding analytical biomarkers, such as creatinine or lactate, or by the use of other parameters that are currently relatively simple to be measured (e.g., SaFi, MAP, GCS), improves our prognostic capacity (17, 32). Indeed, this factor can help in decision making process and in incorporating therapeutic measures that help to improve the prognosis of patients with sepsis at an earlier stage (8).

This study has several limitations. Firstly, this is a convenience cohort, taken over a specific period of time, in a very elderly population. To minimize bias, patients from both urban and rural areas have been included uninterruptedly, and although the number of patients in the sample is adequate, the results cannot be generalized. In addition, not all EMS have POCT and there is no prehospital gold standard score to compare with. It should be noted that both SpO2 and FiO2, individually, presented little clinical significance, this should be considered when using these parameters alone; in this sense, according to our results, we suggest using SaFi instead. On the other hand, patients are taken from transfers by the emergency system with an already high level of severity, which means that we cannot generalize the results to primary care or hospital ED. Finally, our study only evaluates short-term mortality (2-day), but it may be interesting to check the behavior of the scores analyzed in the longer term (e.g., 28-days).

In summary, the mSOFA score performs consistently better in terms of 2-day mortality prediction and in the diagnosis of septic-shock than NEWS2 and qSOFA. The use of associated analytical parameters such as creatinine and lactate integrated in an EWS improves the bedside predictive capacity among patients with suspected infection. The results obtained for mSOFA should be validated in other populations in order to recommend its routine use in prehospital care.

## Data availability statement

The raw data supporting the conclusions of this article will be made available by the authors, without undue reservation.

## Ethics statement

The studies involving human participants were reviewed and approved by the Institutional Review Board of Public Health Service reviewed and approved the investigation (reference: PI-049-19/PI-GR-19-1,258). The patients/participants provided their written informed consent to participate in this study.

## Author contributions

LM-G and FM-R conceptualized the project, managed, and coordinated the project, assisted with design of methodology, analyzed the data, and prepared the initial and final drafts of the manuscript. AS-G take responsibility for the data and their analysis. VL, CM, SC, NM, ID, IS, and MC assisted with management and coordination of the project, assisted with design of methodology, and helped review the manuscript. RL-I conceptualized the project and helped review and commented on the initial and final drafts of the manuscript. All authors contributed to the article and approved the submitted version.

## Funding

This work was supported by the Gerencia Regional de Salud, Public Health System of Castilla y León (Spain) [grant numbers GRS 1903/A/19 and GRS 2131/A/20].

## Conflict of interest

The authors declare that the research was conducted in the absence of any commercial or financial relationships that could be construed as a potential conflict of interest.

## Publisher’s note

All claims expressed in this article are solely those of the authors and do not necessarily represent those of their affiliated organizations, or those of the publisher, the editors and the reviewers. Any product that may be evaluated in this article, or claim that may be made by its manufacturer, is not guaranteed or endorsed by the publisher.

## Footnotes

^1^ http://www.R-project.org
